# Analysis of Factors Hindering the Dissemination of Medical Information Standards

**DOI:** 10.3390/healthcare10071248

**Published:** 2022-07-04

**Authors:** Masami Mukai, Katsuhiko Ogasawara

**Affiliations:** 1 Graduate School of Health Sciences, Hokkaido University, Sapporo 060-0812, Japan; mmukai@ncc.go.jp; 2 Division of Medical Informatics, National Cancer Center Hospital, Tokyo 104-0045, Japan; 3 Faculty of Health Sciences, Hokkaido University, Sapporo 060-0812, Japan

**Keywords:** real-world data (RWD), standards, ISM, DEMATEL, inhabitant factor

## Abstract

Many medical information standards are not widely used in Japan, and this hinders the promotion of the use of real-world data. However, the complex intertwining of many factors hindering the dissemination of medical information standards makes it difficult to solve this problem. This study analyzed and visualized relationships among factors that inhibit the dissemination of medical information standards. Five medical informatics experts affiliated with universities and hospitals were interviewed about the factors that hinder the dissemination of medical information standards in Japan. The presented factors were analyzed using the interpretive structural modeling (ISM) method and the decision-making trial and evaluation laboratory (DEMATEL) method. We found that “legislation” and “reliability” were important inhibiting factors for the dissemination of medical information standards in Japan. We also found a six-layered structure in which “reliability” was satisfied when “legislation” was in place and “expectations” and “personal information” were resolved. The DEMATEL analysis indicated the relationships and classifications of factors hindering the dissemination of medical information standards. Since the adoption of medical information standards does not directly lead to revenue for medical institutions, it is possible to meet the “expectation” of improving the quality of medical care by ensuring “legislation” and “reliability”, that is, ensuring the dependability of medical treatment. The results of this study visually show the structure of the factors and will help solve the problems that hinder the effective and efficient spread of standards. Solving these problems may support the efficient use of real-world data.

## 1. Introduction

In recent years, the use of real-world data (RWD) has been promoted in the medical information domain [1,2,3,4,5,6]. Here, RWD refers to medical data generated by daily medical care, including treatment and medication histories, specimen and physiological function test results, radiology and pathology diagnostic reports, text data from articles written by physicians and nurses, and medical image data from radiological exams. Obtaining new findings by analyzing these medical data is called the development of real-world evidence. For example, in the field of oncology, the FDA approved the expanded indication of Palbociclib in male breast cancer by Flatiron in North America in 2019 [7].

The main purpose of medical information, the source of RWD in hospitals, is to accurately describe and store patient records [8]. Therefore, Yamamoto [9] and Sakai et al. [10] reported that the rules for describing records, assuming secondary use in so-called clinical research, and the standardization of codes to identify drug and test items have not been established in medical institutions, nor have they been thoroughly understood. By establishing an appropriate data quality management system within a hospital, analysis using quality-assured, high-quality RWD can be performed more efficiently and accurately. This is expected to directly improve the accuracy and efficiency of observational studies using medical information and the probability of clinical trial success by enabling the accurate construction of valid hypotheses. However, when we demonstrated the collection of medical data for data use, we found that data collection methods are not unified, and most of the collected data (i.e., drug and specimen test results) are provided in local codes; therefore, a great deal of effort is required to maintain these codes [11,12,13,14,15,16]. In many cases, standards for medical information, such as drug and laboratory test codes, are not applied, even in core clinical research hospitals. The likely reason for this is that digitized coded data are only used in hospitals, while outside hospitals, the data are printed on paper, such as on test result sheets and prescriptions, so there is no particular need for code. Moreover, data integration cannot be undertaken immediately using RWD, meaning there is an urgent need to promote the use of standards. Therefore, it is necessary to first examine the factors that prevent the dissemination and standardization of medical information when considering the infrastructure required for RWD use from an operational perspective. Currently, according to our research, there are no research reports from Japan or any other country on the spread of the standardization of medical information.

ISM can determine relationships among factors that constitute a system by means of a hierarchical diagram. A multivariate analysis reveals interrelationships among factors through pairwise comparison, and it then structures each factor by performing logical operations on the results. This method can be used to visualize a problem comprising many factors as a hierarchical model. The decision-making trial and evaluation laboratory (DEMATEL) method is another structured modeling method that expresses the strength and importance of relationships between the factors that constitute a system. A system is defined as “a set of factors and a set of relationships defined by the set of factors” and interrelationships that are expressed by direct and indirect effects and causal relationships. The interrelationships are determined by pairwise comparisons between factors, and they are expressed as a relational matrix. The sum of rows of the relationship matrix (total influence matrix) represent the degree of influence of each factor in the interrelationship structure. The sum of columns expresses the strength of a factor’s affected degree in the interrelationship structure, which is called the affected degree. The sum of the influence degree and the effect degree is called the centrality, and the value obtained by subtracting the affected degree from the influence degree is called the causality. In medicine, ISM has been used by Thakur et al. to study the efficient and safe disposal of medical waste, by Sarikhani et al. to study the components of hidden cubiculum in the field of healthcare education, and by Bahadori et al. to study the process of medical institution management strategy formulation. Additionally, the DEMATEL method has also been used to identify factors that undermine medical safety [17,18,19,20,21,22,23,24,25,26,27,28,29,30] and the analysis of barriers to the implementation of public health and social measures to prevent COVID-19 infection [31]. However, DEMATEL has not been used to disseminate medical information standards or examine strategies for doing so.

Therefore, the purpose of this study was to identify the factors that hinder the dissemination of standards in the medical information field. By identifying the inhibiting factors, it is expected that measures for disseminating standards can be taken.

## 2. Materials and Methods

**Figure 1:**
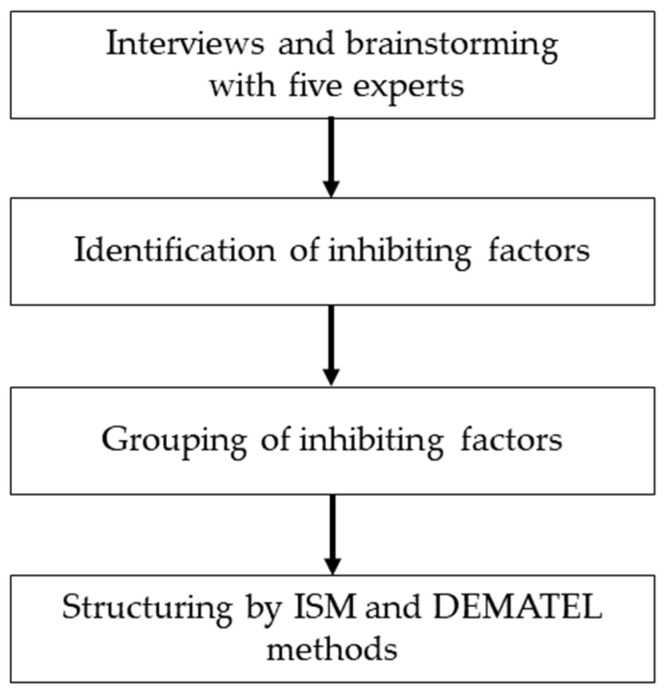
Flowchart of the analysis steps.

We used ISM and the DEMATEL method to identify the structure of factors that inhibit the dissemination of medical information standards. To extract the factors, the target “standard” was the Ministry of Health, Labour and Welfare (MHLW). We defined the system as “inhibiting factors for the dissemination of standardization of medical information”. A flowchart of this study’s analytical steps is presented in Figure 1.

### 2.1. Extraction of Factors through Interviews

The factors that inhibit the spread of the standardization of medical information were extracted from interviews with five experts in medical informatics at universities and hospitals: an associate professor at a national university with 20 years of experience in medical informatics, a lecturer at a national university with 15 years of experience, an associate professor at a private university with 25 years of experience, a researcher at a government agency with 15 years of experience, and a researcher at a national research organization with 23 years of experience. The experts were selected for their expertise in hospital information systems and clinical radiology, regional coordination systems, database engineering and IT, public health, IT, security, and statistics. The interviews were conducted by requesting that each expert send their responses to the administrators directly so that the opinions of the other experts were not influenced.

### 2.2. Extraction and Grouping of Dissemination Inhibiting Factors

Factors considered to be common among those presented in the interviews were sorted and categorized. In classifying the factors, the administrator (a researcher at a government agency with 15 years of experience) checked all of the responses, grouped them, gave each group a name to avoid confusion in the following analysis, and defined them as factors. The defined factors were reviewed by another expert in the field of medical informatics (a professor at a national university with 30 years of experience in medical informatics) and were confirmed.

### 2.3. ISM and DEMATEL Methods

A relational matrix was created by taking two of the defined factors as elements and expressing the presence or absence of a causal relationship between them as 1 and 0, respectively. The relational matrix was reviewed and finalized by the administrator (a researcher at a government agency with 15 years of medical informatics experience) and reviewed by another expert in the field of medical informatics (a professor at a national university with 30 years of medical informatics experience). The relationships between all the elements was then derived using binary Boolean algebra, and the relational structure was expressed in the form of a hierarchical directed graph.

The following binary Boolean algebra arithmetic expression was used:

(A + I)^r−1^ ≠ (A + I)^r^ = (A + I)^r+1^ = T (1)

The DEMATEL method created the direct effect matrix D^d^, the strength that provides the effect among the factors, and calculated its inverse matrix to create the total effect matrix T, which includes the indirect effects of those influences. The obtained values (i.e., the influence degree, affected degree, and centrality) are shown in the graph.

M = D(I − D) ^−1^ (2)

where M is the total effect matrix, D^d^ is the direct effect matrix, D is the normalized direct effect matrix, and I is the unit matrix.

## 3. Results

### 3.1. Extraction of Factors from Interviews and a Summary of Elements

A total of 125 factors were identified in the interviews with the five medical informatics experts. The classified results were defined as dissemination-inhibiting factors. Table 1 presents some of these extracted factors, and Table 2 lists the defined factors.

### 3.2. ISM

**Figure 2:**
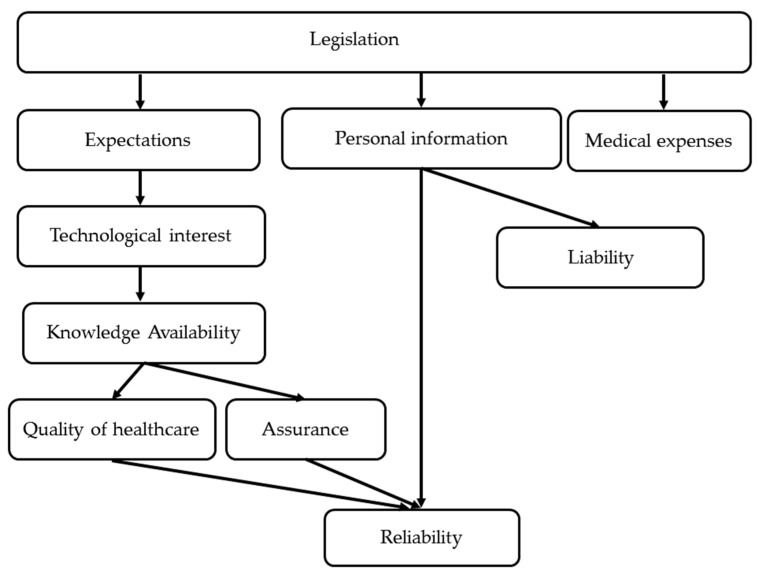
Interpretive structural modeling hierarchy diagram.

The results of the ISM were classified into six tiers, with “legislation” at the top level, as shown in Table 3 and Figure 2.

“Legislation” was the most fundamental factor for inhibiting the spread of the standardization of medical information, followed by “expectations,” “personal information,” and “medical expenses.”

“Reliability” was at the bottom of the list, and two relationships were found to guarantee this reliability: (i) “expectations”—“quality of healthcare” and “assurance”, and (ii) “personal information.”

### 3.3. DEMATEL

**Figure 3:**
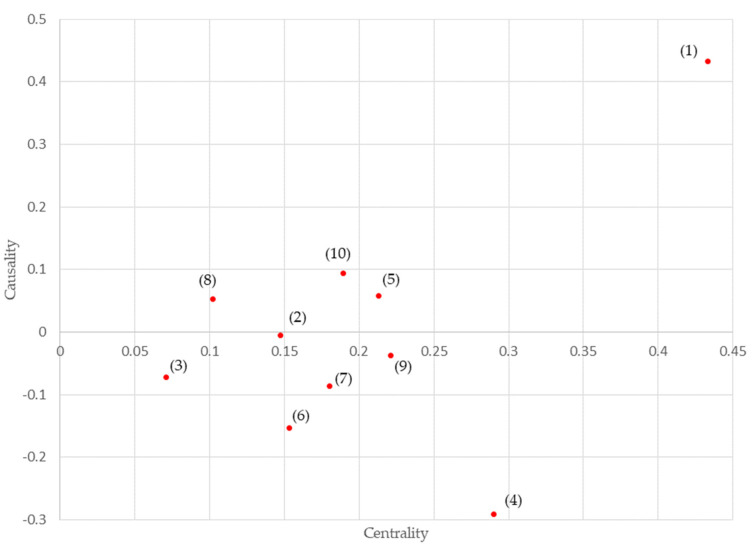
Graphs with Centrality and causality of elements identified by the decision-making trial and evaluation laboratory method. The meanings of the numbers in the figure are shown in Table 4.

The results of the DEMATEL method are shown in Table 4 and Figure 3. The results show that “legislation” (1) had the highest degree of causality and centrality, while “reliability” (4) had a high degree of centrality but the lowest degree of causality. Both factors were located at the top and bottom of the ISM hierarchical structure diagram, consistent with the results of the DEMATEL method for the degree of causality. In addition, both “legislation” (1) and “reliability” (4) had high centrality in the system.

In the ISM hierarchical structure, these two elements were in the upper and middle ranks, but they were attached to other elements because they were not preceded by any other element. In the elements from “expectations” to “quality of healthcare” and “assurance” in the ISM hierarchical structure chart, “expectations” (8) and “technological interest” (5) had almost the same level of causality, with the latter having higher centrality.

## 4. Discussion

Using ISM, we determined that the factors that inhibit the standardization of dissemination are, in ascending order, “legislation,” “expectations,” “personal information,” and “medical expenses,” which is consistent with the current situation whereby medical care is provided based on legislation. It is likely that “expectations” was extracted because the scope of medical information standardization will be determined, and the sense of expectation will increase when it is clearly stated in law.

“Reliability” being placed at the lowest level indicates that the reliability of the standardization of medical information is ensured when other factors are in place. In addition, two points were mentioned in relation to “reliability”: “expectations” due to “quality of healthcare” and “assurance”, and “personal information.” The former relationship is largely dependent on medical professionals. “Expectations” lead to “knowledge availability” through “technological interest” in the standardization of medical information, and “quality of healthcare” and “assurance” are realized by turning knowledge into practice. However, the latter is largely dependent on the government. Specifically, by identifying the scope and risks of handling “personal information” through “legislation” in standardizing medical information, personal information leads to “reliability,” handled in accordance with government rules. This point will be discussed further using the example of the digital imaging and communications in medicine (DICOM) standard.

The high centrality of “legislation” (1) and “reliability” (4) in the DEMATEL results indicates that these are the two most important factors in the system of “factors inhibiting the spread of the standardization of medical information.” However, this likely represents the fact that progress in “legislation” (1) will ensure “reliability” (10).

As for the other elements, “medical expense” (3) and “liability” (6) both had low centrality and causality. Among the “factors inhibiting the spread of the standardization of medical information,” these relied on other elements and were themselves not significant factors.

The ISM located these two elements in the upper and middle ranks of the hierarchical structure, consistent with there being no other elements following them. This was because “medical expenses” (3) are determined by medical fees, and “liability” (6) is realized by determining where the legal responsibility lies concerning personal information and medical practice, consistent with the current medical care system and the scope of work of medical practitioners.

The results of the element of “expectations” regarding “quality of medical care” and “assurance” in the ISM hierarchical structure chart reflected that, although “expectations” and “technological interest” were both elements at the level of awareness and had a high degree of causality, the former referred to awareness of the standardization of the medical information itself, while the latter referred to the awareness of such standardization at a practical level. Therefore, there were differences in the degree of centrality. The motivation for the adoption and implementation of medical information standards was expressed numerically.

The following examples are provided to support this. In Japan, 23 active standards have been established in the Guidelines for Standardization of Medical Information, which is the standard for medical information. Among them are standards used in the medical field, such as HS009 (integration healthcare enterprise potable data for imaging (PDI)), HS011 (DICOM), and HS005 (disease name master). Considering the commonalities of these widespread standards, they were already being used regularly as de facto standards when they were adopted. The reason why PDI and DCOM were de facto standards is that in 2000, when these standards were established, radiological images could be digitized, and there was a demand for sharing image information within hospitals using only electronic data instead of the conventional film operation. Since it was common for multiple manufacturers of image-generating devices to operate at medical institutions, a system was required to integrate the image data generated by each device and refer to it at the site of examination. As such, the DICOM standard was adopted to share image data among multiple systems. The DICOM standard was provided by vendors as a paid option for systems at the time the standard was established, but it is now a standard feature of radiological equipment. The reason why medical institutions adopted the DICOM standard and installed it in their systems, despite it being optional, was partly because there were no other standards available at the time, but also because the law allowed certain incentives for the electronic storage of image data with an “additional fee for electronic image management” under medical fees. This is consistent with the results of the ISM and DEMATEL method analyses, in which the most important factor was “legislation” (1). PDI is a protocol used for the exchange of image data (including medical data) on portable media between medical institutions, and this standard became available when the MHLW notified that image data could be provided in an electronic form and on portable media.

In addition, medical institutions have implemented a function in their hospital information systems to select target disease names from the disease name code master because medical institutions are required to specify such names and ICD-10 disease name codes in the medical fees (including DPC applications) that they prepare to generate income.

Currently, standards for medical information that fall into the category of those not being promoted include drug codes and specimen test codes. These standards have not caused any problems in medical practice because they operate within medical institutions, and they have not had any impact on hospital revenues. This is thought to be the reason why these standards have not been widely used compared to disease name codes. This point is also consistent with the results of our analysis.

In the future, when the construction of an environment for the use of RWD is fully developed, it will be necessary to share, collect, and integrate medical data among multiple medical institutions. Since information on medication and specimen test results will be required for data collection, the institutionalization of the assignment of standard codes for data provision and the motivation to adopt standards, for example, the provision of incentives, are expected to promote the spread of these standards. Other standards that have not spread include those related to documents (i.e., discharge summaries and medical information forms). These are still supposed to be performed on paper, and the legislation for electronic operation is likely insufficient. In addition, there are some points that do not fit various use cases in the medical practice and thus fall under “reliability” (4) in the inhibiting factors. This indicates that there are cases where the standard itself should be reviewed to ensure that the use of the standard provides sufficient benefits in terms of both operational and medical safety. As described by Booth et al. [32], this is attracting attention in terms of solving clinical and policy issues using RWD; however, it is consistent with the view that it should not be immediately used for clinical trials in terms of data quality. To create and use real-world evidence, it is essential to ensure data reliability, and this is the cornerstone of integrating and analyzing varied medical information. Data reliability will likely be improved by enabling data use (without processing) from the data source through the spread of the standardization of medical information. However, as pointed out by Feinberg et al. [4], although the creation of real-world evidence has become possible, the issue remains that the efficacy of cancer drugs has not yet been proven [3]. The RWD used by Flatiron [32] is generated from structured data entry by a large number of abstractors using a proprietary data entry tool. This shows that RWD is currently insufficient for the confirmation of drug efficacy because a set of data items (data set) has not been established. This is consistent with the need for “assurance” to ensure the “reliability” of the ISM analysis and, fundamentally, with the need for “legislation.” We predict that not only the current dissemination of standardization but also a discussion of the standard itself will be necessary due to demand, as in the case of standards, such as structured data sets.

In this study, factors inhibiting the spread of the standardization of medical information were identified and associated by brainstorming among five experts affiliated with universities and hospitals in Japan. A limitation of this research is that brainstorming involves subjective factors, and the factors and association results may have varied depending on the areas of expertise and positions of the contributing experts.

## 5. Conclusions

(1) We structured the factors inhibiting the dissemination of medical information standards and determined the significance of the factors using ISM (interpretive structural modeling) and the DEMATEL (decision-making trial and evaluation laboratory) method, respectively.
(2) The results showed that “legislation” and “reliability” were important inhibiting factors for the dissemination of medical information standards.

## Figures and Tables

**Table 1:** Extracted factors (excerpts).

Experts	Factors
Administrative institutions and researchers	No incentive for standardization No regulations or penalties for non-standardized systems No direct benefit to small hospitals Low compatibility with existing systems Data structure differs from facility to facility due to operational differences The significance of the secondary use of medical information is not understood by clinical staff Some products are not standardized at the vendor level Standardized product costs are high (in many cases, they are optional) The optimal data granularity differs between standardization standards and medical institutions Limited situations of medical collaboration using personal information data The benefits of standardization from the viewpoint of medical institutions are not well recognized Low awareness of the initiative among the Ministry of Health, Labour and Welfare, academic societies, and council initiatives Cases where there are no personnel within the medical institution or vendor who can promote standardization Lack of activities to promote the standardization of medical information
National research institute and researchers	Vendors’ responses are mixed Costly (expensive) to deal with and not linked to income, such as medical fees Servers (SS-MIX server) may be necessary Complexity of the master maintenance Difficult to deal with when there is no one dedicated to supporting the system There are many standard codes depending on the field, making it difficult to understand Unclear to what extent one should respond User indifference No incentive for standardization

**Table 2:** Definition of elements.

Elements	Definition (Contents)
Legislation	The need for legislation and guideline maintenance as an environment when using standardization technology in medicine
Quality of healthcare	Expectations for the improvement of medical care quality and treatment through standardized technology or assurance of medical care quality
Medical expenses	The impact of standardized technology use on healthcare costs, or bearing the costs for medical care and treatment using technology
Reliability	Ensuring confidence in medical and medical practice based on standardized technology and its methods
Technological interest	Interest in and understanding of standardization technologies and the means to increase this interest
Liability	Organizing the breakdown of responsibilities for medical care and treatment that use standardized technology
Assurance	Assurance that the system reflects standardized technology, technical response to the fact that the standardized technology itself is being updated, and organization of the maintenance scope
Expectations	Improvement of the medical care quality by the use of standardization technology in medical care, and motivation to use standardization technology in medical care
Knowledge availability	Understanding the standardization technology, and experience of examples of implementation in other fields and its necessity
Personal information	Organizing the handling of personal information as data when developing standardization technologies, and the risks to personal and private information when using standardization technologies

**Table 3:** Interpretive structural modeling relational matrix.

	Legislation	Quality of Healthcare	Medical Expenses	Reliability	Technological Interest	Liability	Assurance	Expectations	Knowledge Availability	Personal Information
**Legislation**	0	0	3	0	1	4	4	1	0	2
**Quality of healthcare**	0	0	0	3	0	0	0	0	0	0
**Medical expenses**	0	0	0	0	0	0	0	0	0	0
**Reliability**	0	0	0	0	0	0	0	0	0	0
**Technological interest**	0	0	0	0	0	0	0	0	4	0
**Liability**	0	0	0	0	0	0	0	0	0	0
**Assurance**	0	0	0	2	0	0	0	0	0	0
**Expectations**	0	0	0	0	2	0	0	0	0	0
**Knowledge availability**	0	2	0	0	0	0	1	0	0	0
**Personal information**	0	0	0	4	0	2	0	0	0	0

**Table 4:** Indicators of inhibiting factors identified by the decision-making trial and evaluation laboratory method.

	Inhibition Factor	Affected Degree (a)	Influence Degree (b)	Centrality (a + b)	Causality (b − a)
(1)	legislation	0	0.433	0.433	0.433
(2)	quality of healthcare	0.076	0.071	0.147	−0.005
(3)	medical expenses	0.071	0	0.071	−0.071
(4)	reliability	0.29	0	0.29	−0.29
(5)	technological interest	0.077	0.136	0.213	0.059
(6)	liability	0.153	0	0.153	−0.153
(7)	assurance	0.133	0.047	0.18	−0.086
(8)	expectations	0.024	0.078	0.102	0.054
(9)	knowledge availability	0.129	0.092	0.221	−0.037
(10)	personal information	0.047	0.142	0.189	0.095

## Data Availability

Not applicable.
